# Comprehensive Somatic Copy Number Analysis Using Aqueous Humor Liquid Biopsy for Retinoblastoma

**DOI:** 10.3390/cancers13133340

**Published:** 2021-07-03

**Authors:** Mary E. Kim, Ashley Polski, Liya Xu, Rishvanth K. Prabakar, Chen-Ching Peng, Mark W. Reid, Rachana Shah, Peter Kuhn, David Cobrinik, James Hicks, Jesse L. Berry

**Affiliations:** 1 The Vision Center at Children’s Hospital Los Angeles, Los Angeles, CA 90027, USA; maryekim@usc.edu (M.E.K.); ashley.polski@usc.edu (A.P.); lixu@chla.usc.edu (L.X.); ppeng@chla.usc.edu (C.-C.P.); mreid@chla.usc.edu (M.W.R.); cobrinik@usc.edu (D.C.); 2 USC Roski Eye Institute, Keck School of Medicine of the University of Southern California, Los Angeles, CA 90033, USA; 3 Department of Biological Sciences, Dornsife College of Letters, Arts, and Sciences, University of Southern California, Los Angeles, CA 90007, USA; pkuhn@usc.edu (P.K.); jameshic@usc.edu (J.H.); 4 Department of Molecular and Computational Biology, University of Southern California, Los Angeles, CA 90007, USA; kaliappa@usc.edu; 5 Cancer and Blood Disease Institute at Children’s Hospital Los Angeles, Los Angeles, CA 90027, USA; rachana@usc.edu; 6 Norris Comprehensive Cancer Center, Keck School of Medicine, University of Southern California, Los Angeles, CA 90033, USA; 7 Department of Aerospace and Mechanical Engineering, Viterbi School of Engineering, University of Southern California, Los Angeles, CA 90007, USA; 8 Department of Biomedical Engineering, Viterbi School of Engineering, University of Southern California, Los Angeles, CA 90007, USA; 9 Department of Biochemistry and Molecular Medicine, Keck School of Medicine, University of Southern California, Los Angeles, CA 90033, USA; 10 The Saban Research Institute, Children’s Hospital Los Angeles, Los Angeles, CA 90027, USA

**Keywords:** retinoblastoma, aqueous humor, liquid biopsy, cell-free DNA, circulating tumor DNA, SCNA, prognostic biomarker, precision oncology

## Abstract

**Simple Summary:**

Aqueous humor (AH) liquid biopsy is an enriched source of cell-free circulating tumor-derived DNA for retinoblastoma (RB). The use of this AH liquid biopsy allows for genomic analysis of eyes in the absence of tumor tissue. Development of this platform was critical because direct tumor biopsy is prohibited in RB due to risk of extraocular tumor spread. In this retrospective study, we provide comprehensive, whole-genome analysis of the somatic copy number alterations (SCNAs) in 68 eyes of 64 RB patients. We show that the prevalence of specific SCNAs differ between eyes that required immediate enucleation (surgical removal) and eyes that were attempted to be saved but subsequently failed treatment, requiring secondary enucleation. Increases in chromosomal instability, or higher number of broad genomic alterations, predict higher risk clinical and biomarker features in these eyes. Prospective analyses are needed to further determine the clinical relevance and application of these findings.

**Abstract:**

Aqueous humor (AH) liquid biopsy has been established as a surrogate tumor biopsy for retinoblastoma (RB). Previous AH studies have focused on highly recurrent RB somatic copy number alterations (SCNAs) including gain of 1q, 2p, 6p, and loss of 13q and 16q. In this retrospective study, we provide a comprehensive, whole-genome analysis of RB SCNAs and evaluate associated clinical features for 68 eyes of 64 RB patients from whom AH was obtained between December 2014 and October 2020. Shallow whole-genome sequencing of AH cell-free DNA was performed to assess for SCNAs. The prevalence of specific non-highly recurrent SCNAs, such as 20q gain and 8p loss, differed between primarily and secondarily enucleated eyes. Increases in chromosomal instability predict more advanced seeding morphology (*p* = 0.015); later age of diagnosis (*p* < 0.0001); greater odds of an endophytic tumor growth pattern (without retinal detachment; *p* = 0.047); tumor heights >10 mm (*p* = 0.09); and containing 6p gain, a biomarker of poor ocular prognosis (*p* = 0.004). The AH liquid biopsy platform is a high-yield method of whole-genome RB SCNA analysis, and SCNAs are associated with numerous clinical findings in RB eyes. Prospective analyses are encouraged to further elucidate the clinical relevance of specific SCNAs in RB.

## 1. Introduction

Retinoblastoma (RB), a cancer of the developing retina in infants and toddlers, is the most common pediatric intraocular malignancy [1] and accounts for 1% of childhood cancer mortality [2]. RB tumorigenesis has been extensively studied. In the vast majority of cases analyzed, biallelic inactivation of the tumor suppressor gene *RB1* results in development of a premalignant retinoma [3,4,5,6,7], and additional mutational or epigenetic events may promote progression of a retinoma to fully malignant RB [3,8]. By activating oncogenes and inactivating tumor suppressor genes, somatic copy number alterations (SCNAs) are thought to contribute to subsequent RB progression [3,9,10,11,12,13]. However, some tumors are negative for SCNAs or these genomic alterations are sub-clonal, suggesting a complicated series of heterogenous events for tumorigenesis in RB. As most studies have been done on tissue from enucleated eyes, we know very little about these events in less advanced eyes and additionally what new alterations may form under selective therapeutic pressure during attempts to salvage the eye.

Although highly recurrent RB SCNAs—gains on 1q, 2p, 6p; losses on 13q and 16q; and focal *MYCN* amplification—are well established and documented [10,11,14,15,16], these analyses were performed exclusively on tumor tissue from enucleated eyes. This was due to previous inability to biopsy RB for risk of extraocular seeding [17,18]. Our group addressed this limitation by demonstrating that aqueous humor (AH) can be safely extracted and is a rich source of circulating tumor DNA (ctDNA) within the AH [19,20,21]. Using this platform, we have been able to establish relationships between specific genomic alterations and ocular prognosis that could not previously be evaluated in tumor tissue from enucleated eyes only. For example, by comparing genomic profiles of ctDNA between the AH of eyes that were cured and salvaged versus those that required enucleation, we were able to demonstrate that the highly recurrent RB SCNA 6p gain is a biomarker of poor prognosis, portending an increased risk for treatment failure and enucleation [20,22]. Similarly, focal *MYCN* amplification is known to cause aggressive disease in wildtype *RB1*, and in a small cohort we have shown that it is a biomarker of poor prognosis for globe salvage regardless of *RB1* status [20,22]. This was the first time that any RB SCNA was able to be connected to clinical ocular outcomes for eyes actively undergoing salvage therapy, and this association was facilitated by the AH liquid biopsy platform.

While past studies focused on highly recurrent SCNAs, the aim of the present study was to employ the AH liquid biopsy to comprehensively characterize SCNAs that exist genome-wide in RB eyes actively undergoing treatment. Previous whole-genome analyses of RB SCNAs were limited to tumor tissue from enucleated eyes, so little was known about the genomic landscape of tumors in less advanced, salvaged eyes or new alterations identified during therapy [10,11,14,15,16]. Additionally, although our group has since used the AH liquid biopsy to identify RB SCNAs in both salvaged and enucleated eyes, these studies focused primarily on a limited subset of highly recurrent SCNAs and did not evaluate for other less common SCNAs that might also be contributing to RB tumorigenesis, seeding, treatment failure, or progression of disease [19,20,21,22,23,24,25,26]. Herein we present a comprehensive (whole-genome) analysis of RB SCNAs using the AH liquid biopsy. With this minimally invasive platform, we hope to further elucidate the relationship between SCNAs and RB progression, disease severity, and prognosis for eye salvage.

## 2. Materials and Methods

This research was conducted under Children’s Hospital Los Angeles (CHLA) Institutional Review Board approval and adhered to the tenets of the Declaration of Helsinki. Written informed consent was obtained from the legal guardians of all participants.

This study included all patients diagnosed with RB between December 2014 and October 2020 at CHLA from whom written parental consent and aqueous humor sample(s) were obtained. Hence, this study includes AH samples that have been analyzed and published previously, in addition to new samples taken after our most recent publications. Case numbers remained consistent with prior studies for comparison purposes [20,21,22,25,26]. As in previous studies [19,20,21,22,23,24,25,26], liquid biopsy specimens consisted of approximately 100 µL AH taken from the anterior chamber via clear corneal paracentesis for research purposes only. Treatment of all RB patients was carried out in a non-randomized manner per CHLA protocol [27,28,29], and treating physicians were blinded to the results of AH analyses. In general, treatment courses include six cycles of systemic chemotherapy (carboplatin, etoposide, and vincristine) or intra-arterial chemotherapy with melphalan, followed by local treatment that can range from targeted laser and cryotherapy to intravitreal chemotherapy injections to treat vitreous seeds [27,28,29]. 

The samples evaluated are part of a biorepository of AH samples aimed at understanding whether genomic markers correlate with ocular outcomes and the presence of other clinical features. The timing of the first AH sample for each patient depended on the IRB approval governing our research at the time and was either (1) at diagnosis (9/68, 13%), (2) at the end of systemic chemotherapy (4/68, 6%), (3) end of intra-arterial melphalan treatment (1/68, 1%), (4) with intravitreal melphalan injection (33/68, 49%), (5) with enucleation (19/68, 28%), or (6) with bevacizumab injection (1/68, 1%). Thirty-four eyes (50%) had more than one sample taken over the course of treatment but only the first sample taken was included in this comprehensive analysis, with the exception of case 39 wherein the first AH sample had a tumor fraction below the threshold for SCNA detection (<5%) so the second AH sample was used.

A clinical database regarding clinical presentation, patient outcomes, response to therapy, recurrence, any complications from AH sampling, metastasis, death and follow-up is maintained and updated prospectively, in real time during each examination under anesthesia or clinic visit, per IRB approved protocol; once genomic analyses were completed it was reviewed for clinical information. Information collected from the database herein includes International Intraocular Retinoblastoma Classification (IIRC; a system that classifies tumors confined to the ocular space into five groups of increasing severity from A to E based on clinical features) [30], vitreous seeding morphology, and intraocular tumor growth pattern. Germline *RB1* mutation status was identified per routine clinical leukocyte testing and obtained from the medical record as well; this is not an additional research protocol. Primary clinical endpoints included eye salvage (the ability to save the eye using standard chemotherapeutic modalities) versus enucleation (surgical removal of the eye). Procedure and protocol for primary and secondary enucleation at our institution have been published previously; primary enucleation is done without any other therapy and secondary enucleation is for intraocular relapse after attempts to save the eye [27,28,29]. One eye in this study (Case 48) received one cycle of intra-arterial chemotherapy and developed massive tumor growth less than 1 month from diagnosis; due to the timing, this was classified as primary enucleation.

AH samples were processed and stored according to established protocols that are detailed in previous publications [19,31,32]. Briefly, samples were stored on dry ice immediately following extraction and placed into a −80 °C freezer within an hour after extraction. Samples underwent cell-free DNA (cfDNA) isolation using the QIAamp Circulating Nucleic Acid Kit (Qiagen, Germantown, MD, USA) within 72 h of extraction from the eye (often the same day). CfDNA was constructed into whole genome libraries using the QIAseq Ultralow Input library kit (Qiagen, Germantown, MD, USA) within 72 h of cfDNA isolation. Shallow whole genome sequencing was performed on these constructed libraries at 0.3× depth for copy number profiling within 1 month of library preparation. SCNAs were considered present at 20% deflection from a baseline human genome; the bioinformatics protocols by Baslan et al. used on the AH platform have been previously published [20,31,32].

Genomic instability was calculated as the sum of the absolute values of Log2-transformed expression ratios for all segment bins, excluding chromosome X and Y, and represented as the sum deviation from the median. Higher values indicate greater instability.

Mann–Whitney U, Fisher’s exact, logistic regression, and Poisson regression tests were used to examine the relationship between SCNAs and clinical findings. All analyses were conducted using Stata/SE 14.2 (StataCorp LLC, College Station, TX, USA).

## 3. Results

### 3.1. Patient Demographics and Clinical Outcomes

Sixty-four patients were included in the study; four patients with bilateral disease had both eyes sampled, so a total of 68 eyes were included in the analysis. No patients dropped out or withdrew consent over the study period. Demographics, clinical features, and SCNA findings of all participants are summarized in Figures S1 and S2. The median age at diagnosis for all patients was 15 months. Twenty-nine patients were *RB1* positive (45%), while the remaining 35 patients (55%) were negative for *RB1* germline mutation. Of all eyes, the most commonly diagnosed IIRC group [30] was D (46/68, 68%), although less advanced (Groups B and C) eyes were also included.

Thirty-three eyes were enucleated (49%; 16 primarily; 17 secondarily after chemotherapy) and 35 eyes (51%) were salvaged. No patients had complications secondary to AH sampling, including infection, iris trauma, synechiae, hyphema, or cataract. No child developed extraocular disease or metastatic disease throughout the follow-up period. Clinical follow-up from diagnosis to final evaluation ranged from 6 to 84 months (median, 33.5 months).

### 3.2. Whole-Genome RB SCNA Analysis

**Figure 1:**
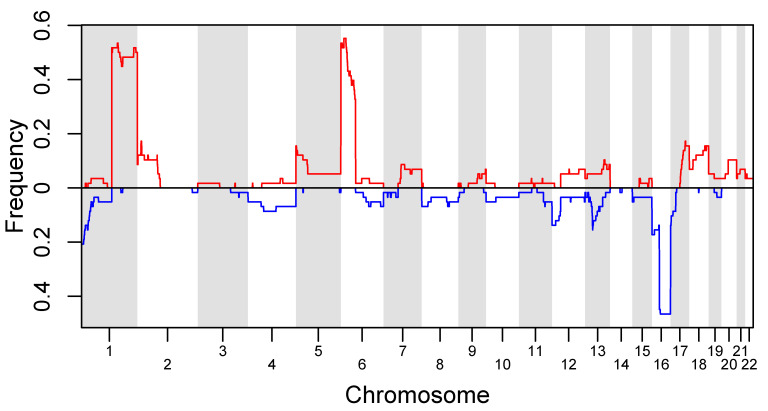
Composite SCNA plot for all eyes included in our study. Gains are represented by the red line and losses are represented by the blue line. Besides previously determined highly recurrent RB SCNAs of 1q, 2p, and 6p gain as well as 16q loss, non-highly recurrent SCNAs were also seen. The most common were 12p loss, 16p loss, 5p gain, 17q gain, 18q gain, 17p loss, 20q gain, and 22p gain.

As in previous studies [19,20,21,22,23,24,25,26], the most common RB SCNAs included highly recurrent 6p gain (33 eyes, 48.5%), 1q gain (33 eyes, 48.5%), 16q loss (28 eyes, 41.2%), 2p gain (11 eyes, 16.2%), and 13q loss (8 eyes, 11.8%). The most common non-highly recurrent RB SCNAs were 12p loss (8 eyes; 11.8%), 16p loss (8 eyes; 11.8%), 5p gain (7 eyes; 10.3%), 17q gain (6 eyes; 8.8%), 18q gain (6 eyes; 8.8%), 17p loss (5 eyes; 7.4%), 20q gain (5 eyes; 7.4%), and 22p gain (5 eyes; 7.4%). Eyes with a 6p gain at or above an amplitude of 1.5 ratio to the median showed significantly more chromosomal instability (mean 507.04, SD 231.95 sum deviation from the median) than eyes without a 6p gain (mean 272.19, SD 263.22 sum deviation from the median; *p* = 0.0002). A composite SCNA frequency plot for all eyes can be seen in Figure 1.

Focal *MYCN* amplification was present in four eyes (6.3%; Cases 3, 10, 31, and 48); the presence of *MYCN* amplification does not necessarily denote a *MYCN*-driven tumor which clearly Case 10 is not (germline *RB1* mutation present). All four of these eyes were enucleated (Table 1). Two eyes (Cases 3 and 48) displayed SCNAs in addition to focal *MYCN* amplification in both the AH and matched tumor tissue, while the other two eyes (Cases 10 and 31) only displayed *MYCN* amplification. There was no somatic *RB1* mutation identified in either the AH or the tumor for any of these cases; on histopathologic review, none of these eyes had characteristic features of primary *MYCN* amplified retinoblastoma tumors [14].

### 3.3. Clinical Correlates of RB SCNAs

We also aimed to evaluate associations between clinical presentation and genomic instability, measured either as whole integer number of SCNAs present or total deviation from median copy number amplitude. In general, eyes with higher chromosomal instability tended to display more advanced clinical disease. Less advanced IIRC Group B eyes had lower genomic instability (*n* = 3, mean 315.90, SD 347.85 sum deviation from the median) than more advanced eyes IIRC Groups C-E, although this was not statistically significant (*n* = 65, mean 338.82, SD 274.02 sum deviation from the median, *z* = −0.34, *p* = 0.73). 

**Figure 2:**
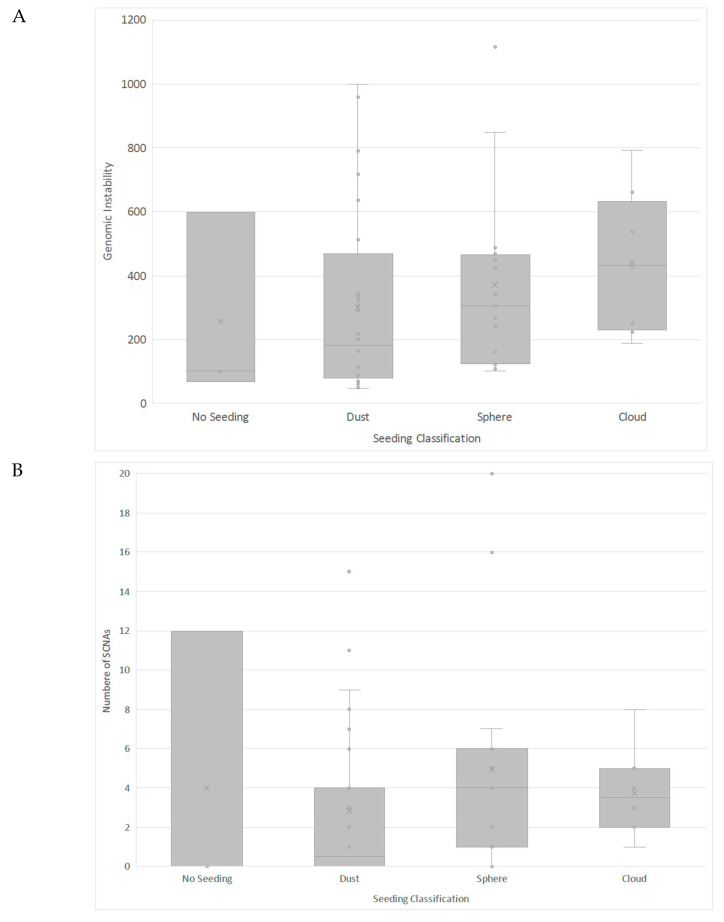
Box and whisker plot showing (**A**) genomic instability based on seeding classification and (**B**) integer numbers of SCNAs based on seeding classification. x indicates the median value.

In terms of seeding, eyes with either dust or no seeding displayed significantly less chromosomal instability compared to eyes with more advanced seeding morphologies of sphere or cloud (*p =* 0.007; Figure 2A). Sphere seeding was associated with the largest whole integer number of SCNAs, followed by cloud, dust and no seeding, but only the comparison between sphere and no seeding was statistically significant (*p* = 0.024; Figure 2B). There was no significant difference in presence or absence of seeding between eyes with or without a 16q loss (*p =* 0.46).

**Figure 3:**
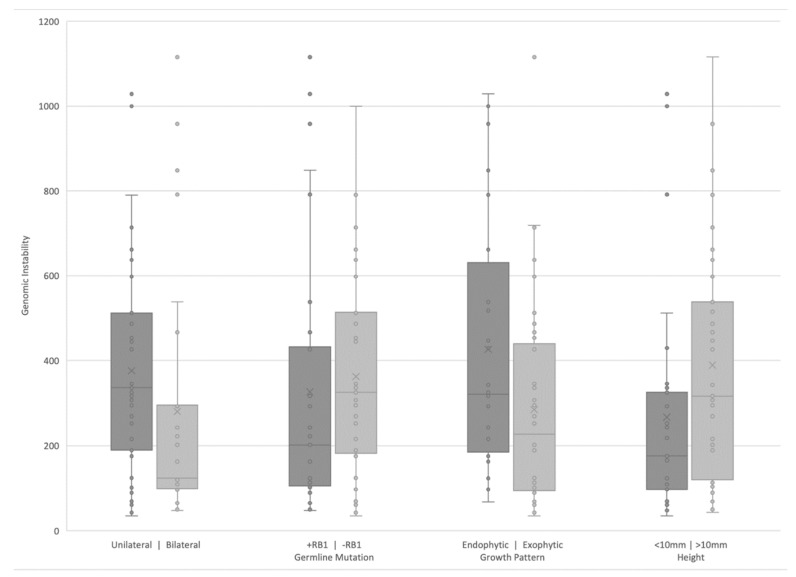
Box and whisker plot showing genomic instability based on laterality, heritability (+ RB1 indicates a germline mutation), growth pattern, and height. x indicates the median value.

As shown in Figure 3, based on laterality, eyes with unilateral RB displayed significantly more chromosomal instability compared to bilateral RB eyes (*p* = 0.03). This corresponded to a difference in age at diagnosis, with unilateral eyes diagnosed at significantly older ages (mean 19.44 months, SD 12.06) compared to bilateral eyes (mean 12.67 months, SD 10.21, *p* = 0.022). When run in Poisson regression, the effects of laterality (IRR = 0.69, 95%CI = 0.40–1.17, *p* = 0.17) and its interaction with age (IRR = 1.01, 95%CI = 0.99–1.04, *p* = 0.20) were non-significant, leaving age at diagnosis as the predictor of chromosomal instability (IRR = 1.04, 95%CI = 1.03–1.05, *p* < 0.001). For each month increase in age at diagnosis, the rate at which SCNAs are detected in patients increases 1.04 times. Similarly, there was no significant difference in the genomic instability based on heritability (presence or absence of *RB1* germline mutation (*p* = 0.23). 

Eyes with an endophytic tumor growth pattern (without retinal detachment) displayed significantly more chromosomal instability than an exophytic growth pattern (*p* = 0.03; Figure 3). However, after controlling for the effects of seeding, the effect was no longer significant (*p* = 0.18). Regarding main tumor size, eyes with initial (pre-treatment) tumor height *≥*10 mm had marginally greater instability than eyes with initial tumor height <10 mm (*p* = 0.05; Figure 3). 

### 3.4. RB SCNAs in Enucleated Eyes

**Figure 4:**
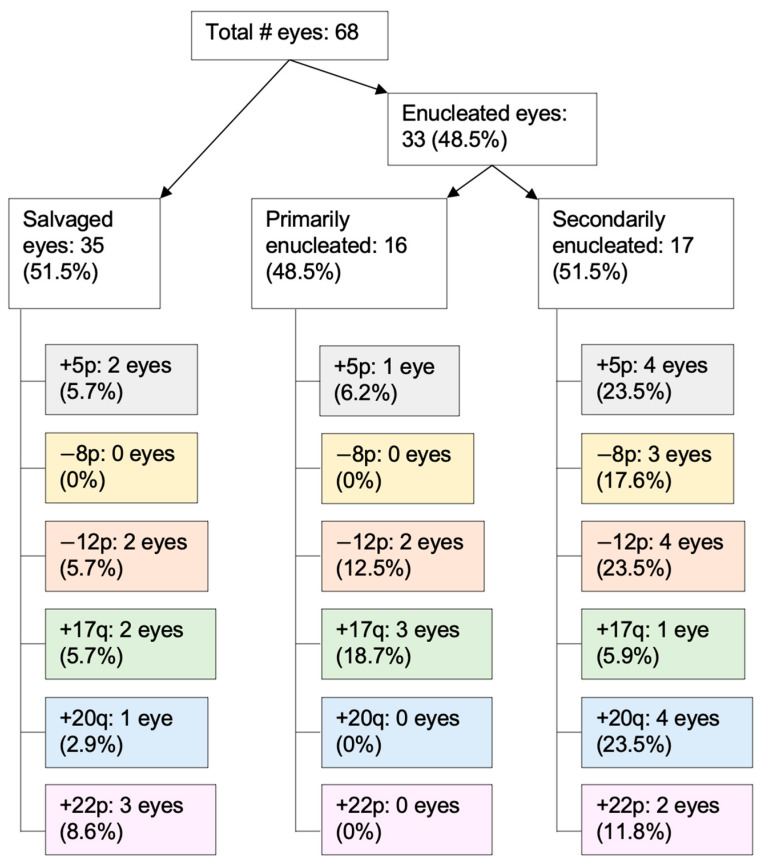
The prevalence of specific non-highly recurrent SCNAs differed between primarily and secondarily enucleated eyes. Note, not all secondarily enucleated eyes had AH sampled prior to enucleation to be used for comparison of new SCNAs.

There was no difference in chromosomal instability between eyes that were treated versus primarily enucleated (treated eyes mean 320.16, SD 295.71 sum deviation from the median; primarily enucleated eyes mean 395.17, SD 186.34 sum deviation from the median; *z* = −1.81, *p* = 0.07). However, the prevalence of specific non-highly recurrent SCNAs does differ between primarily and secondarily enucleated eyes (Figure 4). For example, four eyes demonstrated 20q gain and three eyes demonstrated 8p loss in the secondary enucleation group; these SCNAs were not seen in the primary enucleation group, and this difference approached significance for 20q gain (*p* = 0.10). 

**Figure 5:**
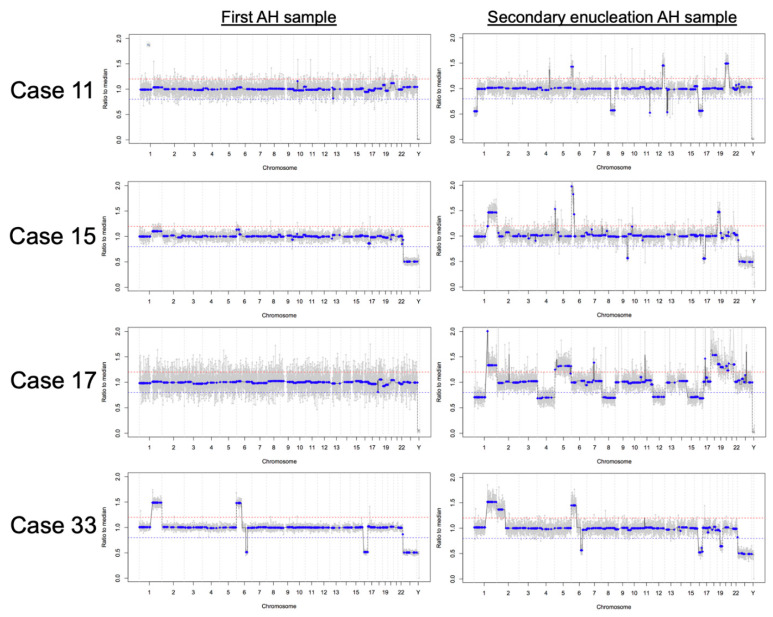
Four eyes demonstrated new SCNAs at secondary enucleation that were not present during conservative management. New SCNAs were as follows: Case 11 (8q loss, 16q loss, 20q gain), Case 15 (1q gain, 6p gain, 17p loss, 19p gain), Case 17 (1p loss, 1q gain, 4p loss, 4q loss, 5p gain, 5q gain, 8p loss, 8q loss, 12p loss, 12q loss, 15q loss, 16p loss, 16q loss, 18p gain, 18q gain, 19p gain, 19q gain, 20p gain, 20q gain, 21q gain), and Case 33 (2p gain, 19q loss). Other focal changes seen are at the centromeres and not true SCNAs. The first samples taken from cases 11, 13, and 17 were during IVM injection, whereas the first sample taken from case 33 was at diagnosis. Treatment courses for these cases are as follows. Case 11 underwent systemic chemotherapy treatment (six cycles of carboplatin, etoposide, and vincristine (CEV)), followed by four IVM injections over three months for recurrent dust seeding; the eye was enucleated 14 months after diagnosis due to primary tumor recurrence. Case 15 began treatment with three monthly intra-arterial melphalan injections, followed by three weekly IVM injections for dust seeding; the eye was enucleated 22 months after diagnosis due to primary tumor recurrence. Case 17 underwent systemic chemotherapy treatment (six cycles CEV), followed by three IVM injections over one month for recurrent sphere seeding; the eye was enucleated two months later due to primary tumor recurrence. Finally, Case 33 was treated with two cycles of CEV as a bridge and three cycles of intra-arterial melphalan, followed by four IVM injections over six weeks to treat persistent dust seeding; the eye was enucleated six months after diagnosis due to apical tumor recurrence with persistently active seeding despite ongoing therapy.

Of secondarily enucleated eyes, six had AH sampled during conservative management and immediately following enucleation. For these six eyes, AH samples obtained at secondary enucleation had a higher number of SCNAs (mean 5.67, SD 7.17) compared to their corresponding AH samples taken during conservative management (mean 1.00, SD 1.26), and this difference approached statistical significance (*p* = 0.055). Four eyes demonstrated new SCNAs at secondary enucleation that were not present in earlier AH samples (Figure 5). The first samples taken from cases 11, 15, and 17 were during IVM injection, whereas the first sample taken from case 33 was at diagnosis.

There was no significant difference in the age at diagnosis of treated versus primarily enucleated eyes (treated eyes mean 15.6 months, SD 12.5; primarily enucleated eyes mean 20.4, SD 8.5; *z* = −1.82, *p* = 0.07).

## 4. Discussion

Herein we present the first comprehensive, whole-genome RB SCNA analysis using the AH liquid biopsy. Although previous genome-wide studies of RB SCNAs were limited exclusively to enucleated tumor tissue [10,11,14,15,16], the AH liquid biopsy for RB has facilitated the investigation of RB SCNAs in all eyes—including those that are actively undergoing therapy or even treatment-naïve [19,20,21,22,23,24,25,26]. The implementation of this organ-specific liquid biopsy allows for evaluation of tumoral information from less advanced eyes (such as Group B eyes), and importantly, with longitudinal sampling of the same eye, the potential to detect impactful new genomic alterations that arise under therapeutic pressure. The findings of this and past studies [19,20,21,22,23,24,25,26] suggest that the AH liquid biopsy is a safe and minimally invasive approach to whole-genome SCNA analysis. With additional larger, prospective studies, results from the AH liquid biopsy may help us better understand the relationships between RB genomics, clinical features, and outcomes.

Although the previously identified highly recurrent RB SCNAs remained the most prevalent among the 68 eyes analyzed here, specific non-highly recurrent RB SCNAs were also seen albeit at far lower rates; this is similar to past publications on tumor tissue [3,4,8,11,33]). Identifying candidate genes in these regions is an important next step given that the ultimate goal of characterizing SCNAs is to uncover their role—if any—in the tumorigenesis, disease severity, and treatment response of RB. This has been done for highly recurrent RB SCNAs in enucleated RB tumor samples [3,4,8,11,33]; however, there have been no candidate gene studies performed on non-surgically removed eyes or non-highly recurrent RB SCNAs, and there is little understanding of the evolution of these SCNAs during treatment as tumoral information was previously unattainable until the time of enucleation. Based on comprehensive analyses of SCNAs in pan-cancer cohorts, there are known cancer driver genes within the non-highly recurrent RB SCNA regions that may contribute to RB tumorigenesis [34,35,36]. For example, within chromosome 5p, amplified in 10% of eyes studied here, the Telomerase Reverse Transcriptase (*TERT*) gene has been identified as an oncogene [34,35,36]. *TERT* is responsible for stabilizing telomere length and when amplified, prevents the permanent growth arrest phase that cells normally enter once telomeres become too short, ultimately leading to uncontrolled proliferation [37,38,39,40]. *TERT* gains have been previously identified in three of 83 enucleated *RB* tumors examined by targeted sequencing [41]. Another candidate gene is Ataxin 2 Binding Protein 1 (*A2BP1*, also known as *RBFOX1*) located in the chromosome 16p [35,36], which was suppressed in 12% of the eyes analyzed here and is frequently a result of whole chromosome 16 loss in RB [11]. *A2BP1* interacts with the known tumor suppressor gene Tropomyosin-1 to promote cytoskeletal organization that leads to terminal differentiation [42,43,44,45,46]. Several genes within the 20q11–13 region have also been identified as potential oncogenes that become amplified in breast and bladder carcinogenesis [47,48] and may play a role in immortalization of human uroepithelial cells [49]. However, the specific role of these genes in cancer development is not fully understood. Candidate gene studies on non-surgically removed RB eyes that include RB SCNAs, other non-highly recurrent SCNAs, and new alterations that arise longitudinally during therapy represent an interesting area of future study using the AH liquid biopsy platform to improve understanding of the broad clinical spectrum of RB.

Comprehensive RB SCNA analysis also provides the opportunity to investigate how genomics relate to clinical features and outcomes. While it is well established that a variety of clinical features affect ocular prognosis [28,30,50,51,52,53], it is not clear whether these clinical features are the result of a genetically ‘high risk’ RB, or if they arise independently of genomics. It bears emphasizing that the clinical characteristics were correlated to genomic alterations in AH samples taken both at time of diagnosis and at later stages throughout treatment. Preliminary data presented herein suggests that clinical features may be associated with RB genomics, however further study is needed before direct causality can be established. In general, eyes with more chromosomal instability displayed higher risk clinical features than eyes with less chromosomal instability. More advanced (IIRC Groups D and E) [30] eyes and eyes diagnosed at older ages tended to have more SCNAs than less advanced eyes and those diagnosed at younger ages. Accordingly, increased age at diagnosis has previously been associated with development of more advanced disease [54]. Although prior studies have reported relationships between chromosomal instability and laterality as well as heritability [6,16,33,55], we postulate that the differences in age at diagnosis, even when controlling for Group classification and heritability, remain the driving factor. This is supported by the finding that any differences in chromosomal stability based on laterality or heritability disappeared when controlling for age at diagnosis. Consistent with previously published work [11,24], this suggests that the number of SCNAs a tumor develops is dependent on the length of time from initial tumorigenesis (biallelic *RB1* loss or *RB1*-null cone precursor proliferation) to diagnosis. Interestingly, eyes with 6p gain, a biomarker of poor ocular prognosis, [20,22] display more SCNAs than eyes without 6p gain, suggesting that once bi-allelic loss of *RB1* initiates genomic instability within a cell, 6p gain may be the first large-scale genomic change that occurs; this may lead to further chromosomal instability. However, the mechanism by which this occurs is not yet clear. Prior studies have identified *E2F3* and *DEK* as candidate genes in the 6p minimum region of gain [3,9,11,20,22]. *DEK* encodes a DNA-binding protein that is a known oncogene in multiple other cancers [56,57], while *E2F3* is crucial for transcriptional cell cycle control and is regulated by the RB protein pRB [58].

In terms of seeding, eyes with cloud or sphere morphologies displayed significantly more chromosomal instability than eyes with dust or no seeding. Cloud and sphere morphologies are higher risk, as eyes with these seeding patterns demonstrate poorer outcomes than those with dust or no seeding [28,53]. Differing chromosomal stability observed among seeding morphologies could be due to their underlying histopathologic differences. Cloud seeds are known to be largely necrotic, thus likely releasing high amounts of ctDNA, while sphere seeds contain several layers of viable tumor cells that can undergo clonal evolution and contribute to higher SCNA counts [59]. However, the seeding morphology a tumor develops could also be driven by RB genomics. We previously demonstrated that each increase in seeding class (from none to dust, from dust to sphere, and sphere to cloud) was significantly associated with two-fold increased odds of having 6p gain, an SCNA that multiple studies have theorized to be an important driver of RB tumorigenesis and aggressive disease [3,20,22]. Although 16q loss has previously been associated with the presence of seeding [60], implicating a functional role for cadherin 13 or cadherin 11 [61] which promotes cellular adhesion, this association with 16q loss was not observed in this comprehensive analysis. This may be due to the fact that enucleated eyes are a different cohort than the eyes included herein and likely have a larger seeding burden than eyes undergoing salvage. Larger studies with the statistical power to control for underlying genomic differences are needed to clarify the relationship between genomics and seeding morphology. 

This comprehensive analysis also revealed that eyes with an endophytic tumor growth pattern (i.e., without retinal detachment) showed more chromosomal instability than eyes with retinal detachment, or with an exophytic tumor growth pattern. This did not remain significant when controlling for seeding, as endophytic RB tumors have a higher risk of seeding into the vitreous than exophytic tumors [62]. In addition to the contribution of seeding, the pathophysiology underlying this finding may be the death of normal retina cells that occurs during retinal detachment; the subsequent lysis of these cells causes the release of cell-free DNA (cfDNA) that is not tumor-derived, thus potentially diluting the tumor fraction below the assay’s sensitivity. However, even if the primary tumor decreases in size, the tumor fraction can remain high in the presence of active seeding [21]. Future study with cfDNA quantification and fragment size analysis is encouraged to further elucidate the relationship between tumor growth pattern and SCNA development. 

Our previously published work showed high concordance between tumor tissue and AH as well as overall stability of AH SCNA profiles within the same eye during intravitreal chemotherapy treatment [20,22]. This comprehensive analysis shows that a small number of eyes gained additional SCNAs seen at the time of secondary enucleation when compared to samples taken from the same eye during earlier conservative management. This increased chromosomal instability at time of secondary enucleation may reflect clonal evolution of the tumor, with newly arising SCNAs potentially indicating genes that proffer treatment resistance and signaling a worsening prognosis for these eyes. However, it is also possible that the tumor fraction in the first AH samples for these eyes (particularly cases 11, 15, and 17, with no SCNAs identified in their first samples which were taken during IVM treatment) was too low for SCNA detection given treatment response, as we have previously demonstrated that low tumor fraction correlates with disease regression [25]. Conversely, we have also shown that approximately one-third of RB tumors do not display SCNAs when AH is sampled during IVM [20,22,25]. Our recent publication evaluated AH samples taken at time of diagnosis [21], with a significantly higher concentration of cfDNA than AH samples taken during treatment, and found that three out of seven eyes demonstrated no detectable large scale SCNAs even at diagnosis. Thus, it may still be that cases 11, 15, and 17 truly did not display any tumoral SCNAs at time of initial AH sampling. With regard to case 33, the first AH sample was taken at time of diagnosis, when ctDNA concentrations are usually highest [21]. The presence of ctDNA in case 33′s first sample is confirmed by the four SCNAs seen in this genomic profile. With this in mind, the additional SCNAs of 2p gain and 19q loss in case 33′s secondary enucleation sample offer future targets of study for molecular treatment resistance. There were also differences in SCNAs between primarily and secondarily enucleated eyes, which may suggest alterations conferred during the course of therapy. In particular, 8p loss and 20q gain, seen almost exclusively in secondarily enucleated eyes, represent interesting targets of study for treatment resistance genes if proven to be present at statistically significant increased rates (Figure 3). Alterations in chromosome 8 are rarely reported in the literature, but 20q gain has been seen both in RB [16,63] and other cancers [64]. Further studies with longitudinal evaluation of the AH ctDNA from diagnosis throughout treatment will allow us to better clarify these findings.

*MYCN* driven tumors remain of interest to the RB community, given that they are described to be aggressive and non-responsive to treatment particularly in the setting of wild type *RB1* (i.e., no *RB1* mutation) [14,65]. In this study population, we present four eyes (Cases 3, 10, 31, and 48) with *MYCN* amplification identified; all four eyes were enucleated. Focal *MYCN* amplification in the setting of biallelic *RB1* inactivation has been previously described [41,65,66,67,68] and can be seen in case 10 of this study population (germline *RB1* mutation). Although additional SCNAs in *MYCN* driven tumors are rare [14,69], cases 3 and 48 displayed highly recurrent RB SCNAS (plus 16p loss in case 3) in addition to *MYCN* amplification. Longitudinal samples within more *MYCN* eyes would be helpful to better understand the genomic basis of both the rare, primarily *MYCN*-driven tumors in the setting of wild-type RB, as well as ‘secondary’ *MYCN* amplification in the setting of *RB1* loss. This includes whether the development of additional SCNAs portends any increased risk to these eyes. Regardless, the potential utility of applying the AH liquid biopsy to identifying *MYCN* driven tumors at diagnosis remains clear.

## 5. Conclusions

We present the first comprehensive, genome-wide analysis of RB eyes using the AH liquid biopsy, along with a preliminary investigation into the relationships between RB genomics, clinical features, and outcomes. Compared to past publications, identification of these alterations via the AH liquid biopsy platform allows for a more comprehensive analysis as it includes not only enucleated eyes, but also less advanced eyes that have been salvaged and longitudinal evaluation during therapy. Furthermore, this allows for the detection of changes longitudinally during treatment such as new alterations at the time of secondary enucleation. In general, eyes with high chromosomal instability tended to have more advanced seeding morphologies, significantly larger tumor heights, significantly older ages at diagnosis, and were significantly more likely to demonstrate 6p gain, a biomarker of poor ocular prognosis. We emphasize that further studies—including more eyes and across multiple treatment centers—are needed before the AH liquid biopsy can be clinically applied and in order to clarify many of the associations reported here with statistical significance. In addition to advancing understanding of the RB genomic landscape, potential applications of the AH liquid biopsy include guiding clinical decision making and the opportunity to provide precision cancer care to RB patients in the future.

## 6. Patents

Drs. Berry, Xu, and Hicks have filed a patent application entitled, Aqueous Humor Cell Free DNA for Diagnostic and Prognostic Evaluation of Ophthalmic Disease.

## Figures and Tables

**Table 1:** Clinical and genomic characteristics of *MYCN* amplification tumors.

Case	Laterality	Age at Diagnosis (months)	Germline *RB1* Mutation	Timing of Enucleation	Other SCNAs	Somatic *RB1* Mutation in AH	Somatic *RB1* Mutation inTumor	Distinct Histologic Features for *MYCN* Amplified Tumors *
3	Unilateral	38	Negative	Primary	1q and 6p gain, 16p and 16q loss	None	None	None
10	Bilateral	2	Positive	Secondary for persistent seeding	None	None	None	None
31	Unilateral	9	Negative	Primary	None	None	None	None
48	Unilateral	18	Negative	Primary	1q gain	None	None	None

* distinct histologic features in *MYCN*-amplified tumors include large prominent undifferentiated cells with multiple nucleoli, necrosis, apoptosis, and minimal calcification [14].

## Data Availability

The data presented in this study will be available on request from the corresponding author. Due to NIH funding, the data will be available to other researchers via NIH GDS/dbGAP controlled databases and will also be available to the public upon request from the corresponding author.

## Supplementary Materials

The following are available online at https://www.mdpi.com/article/10.3390/cancers13133340/s1, Figure S1: Demographic and Diagnostic Patient Information, Figure S2: Genomic Information.
