# Supplementary material for: Comprehensive Somatic Copy Number Analysis Using Aqueous Humor Liquid Biopsy for Retinoblastoma
Source: Cancers (Basel). 2021 Jul 3;13(13):3340. doi: 10.3390/cancers13133340 (PMC8268955; doi:10.3390/cancers13133340)
Supplement: Supplementary file 1 [file cancers-13-03340-s001.zip › cancers-1261254-supplementary.pdf]

cancers

MDPI

Supplementary Material: Comprehensive Somatic Copy Number Analysis Using Aqueous Humor Liquid Biopsy for Retinoblastoma

Mary E. Kim, Ashley Polski, Liya Xu, Rishvanth K. Prabakar, Chen-Ching Peng, Mark W. Reid, Rachana Shah, Peter Kuhn, David Cobrinik, James Hicks and Jesse L. Berry

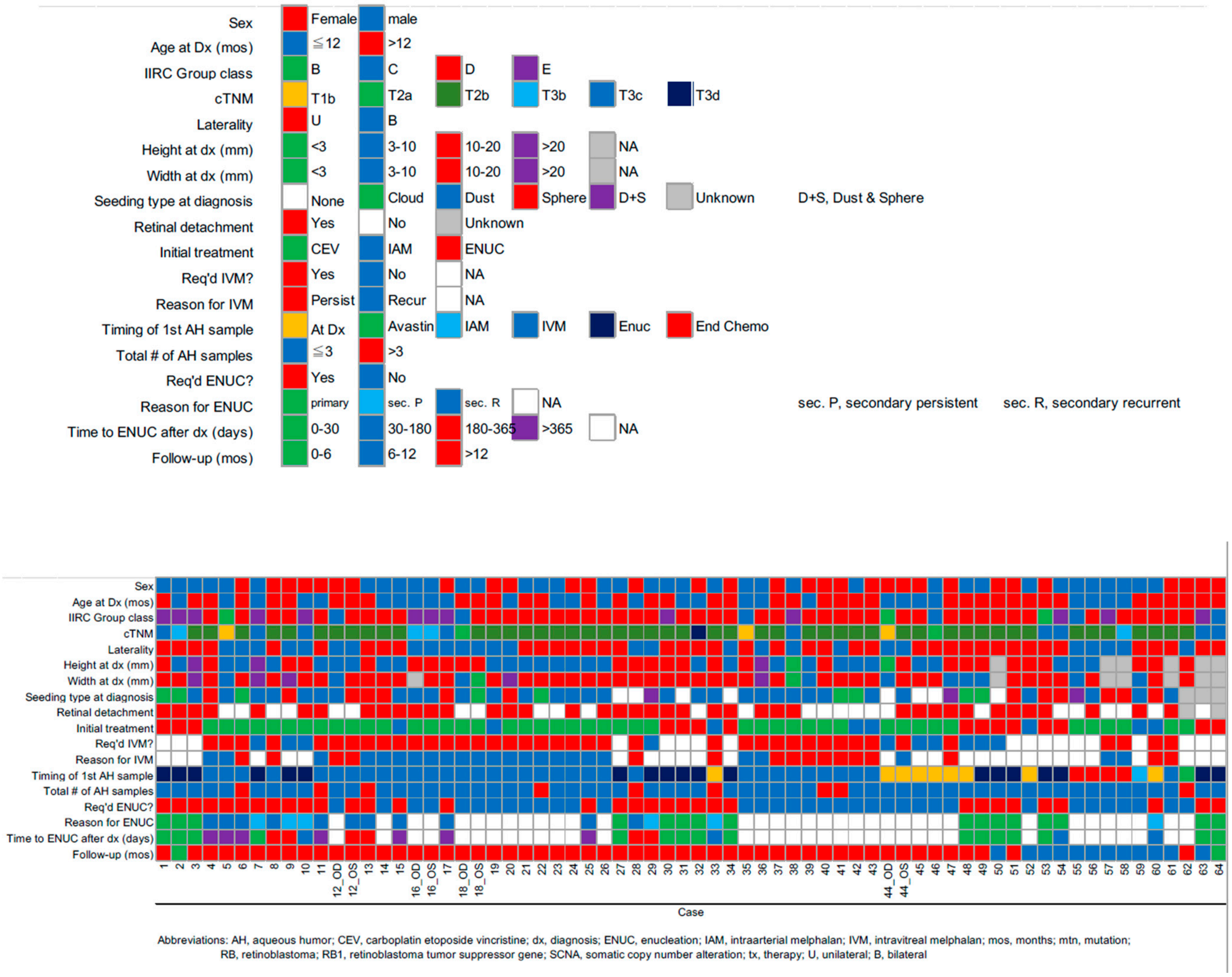

Figure S1. Demographic and Diagnostic Patient Information.

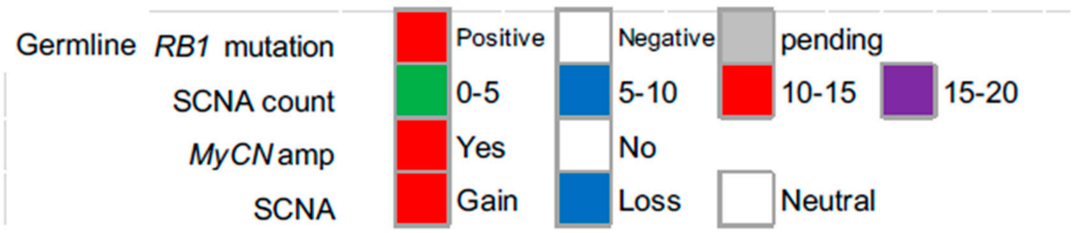

Figure S2. Genomic Information.
